# The Relationship Between Postrelease Social Determinants of Health and Glycemic Control in African Americans With Criminal Legal Involvement and Type 2 Diabetes

**DOI:** 10.1155/jdr/3650434

**Published:** 2026-06-11

**Authors:** Olaitan Akinboboye, Rebekah J. Walker, Jennifer A. Campbell, Laura Hawks, Joni S. Williams, Leonard E. Egede

**Affiliations:** ^1^ Department of Family and Community Medicine, Western Michigan University, Kalamazoo, Michigan, USA, wmich.edu; ^2^ Division of Population Health, Department of Medicine, Jacobs School of Medicine and Biomedical Sciences, University of Buffalo, Buffalo, New York, USA, buffalo.edu; ^3^ Division of General Internal Medicine, Department of Medicine, Medical College of Wisconsin, Milwaukee, Wisconsin, USA, mcw.edu; ^4^ Center for Advancing Population Science (CAPS), Medical College of Wisconsin, Milwaukee, Wisconsin, USA, mcw.edu

**Keywords:** glycemic control, postrelease, social determinants, Type 2 diabetes

## Abstract

**Background:**

Limited literature exists on the relationship between postrelease social determinants of health and glycemic control among African Americans with a history of criminal legal involvement and Type 2 diabetes mellitus (T2DM).

**Methods:**

Participants were recruited from clinics, community centers, and places of worship in the midwestern United States. Stepwise regression with forward selection was used to evaluate the relationship between postrelease criminal justice system factors, social risk/environmental factors, and glycemic control among African American with history of criminal legal involvement and T2DM.

**Results:**

Retained variables from the stepwise regression included age, comorbidity, type of medication, length of incarceration, insurance, and housing insecurity. The analysis revealed statistically significant associations between comorbidity, type of medication, and insurance type with glycemic control. Compared with using only pills or oral hypoglycemic agents, the combination of pills/oral hypoglycemic agents and insulin (*β*‐coefficient: 1.44; 95% CI: 0.77, 2.12) and using insulin or injectables alone (*β*‐coefficient: 0.95; 95% CI: 0.16, 1.73) were associated with an increase in HbA1c. Additionally, each additional comorbidity (*β*‐coefficient: −0.25; 95% CI: −0.40, −0.11) was associated with a decrease in HbA1c. Compared with individuals with private/military/other insurance, those with Medicare (*β*‐coefficient: 1.40; 95% CI: 0.43, 2.37) and Medicaid (*β*‐coefficient: 1.11; 95% CI: 0.12, 2.10) were associated with higher HbA1c.

**Conclusion:**

Postrelease criminal justice factors and social/environmental risk factors were not statistically significant. Future studies should evaluate the relationship between insurance type and glycemic control among this population.

## 1. Introduction

Diabetes mellitus, the eighth‐leading cause of death in the United States [1], is a condition of significant public health concern. Its incidence in the United States has been sharply increasing, rising from 7.4% in 2015 to 11.6% in 2021 [1, 2], with 1.2 million individuals in the United States newly diagnosed every year [1]. Moreover, there are substantial disparities between African Americans and White Americans in both prevalence of and complications from diabetes. The prevalence of diabetes among African Americans is 12.1% compared with 6.9% for non‐Hispanic White Americans [2], and African Americans are two times more likely to die from diabetes and its complications compared with White Americans [3]. The reasons for racial disparities in diabetes burden are not fully known and likely multifactorial, though evidence shows that unequal exposure to social risk factors plays a major role [4, 5].

Social determinants of health (SDOH) represent upstream structural and contextual conditions shaped by economic, environmental, and policy systems that influence patterns of health and disease across the life course [6]. According to the World Health Organization (WHO), socioeconomic position and social status result from the interplay of political, economic, and cultural factors, influencing various SDOH components like behaviors, biology, psychosocial factors, and environment [7]. Research links SDOH to diabetes outcomes [4, 8, 9]; for example, US individuals with diabetes and no college education exhibit poorer outcomes than those with some college education [10]. Similarly, a longitudinal study associates financial hardship with suboptimal glycemic control [8]. The SDOH describe domains that influence health outcomes; social risk factors describe unfavorable conditions at the individual level that impact health outcomes and accentuate health disparities [10]. One less‐explored social risk factor in relation to chronic disease outcomes is exposure to incarceration [11, 12].

Incarceration stands as a social risk exposure with long‐term downstream effects on health behaviors, healthcare access, and chronic disease management [13]. A parallel can be drawn between the disparities in diabetes prevalence and mortality rates among different racial groups, as African Americans confront higher incarceration rates compared with their white counterparts [14]. On an annual basis, over 600,000 individuals are released from prisons, with approximately 9 million cycling through local jails [15]. Literature widely acknowledges the challenges confronted by those with a history of legal involvement [15, 16]. Re‐entry into community life is often complicated by structural barriers, disrupted social networks, and persistent stigma linked to prior justice system involvement [17]. Securing and maintaining employment becomes a formidable challenge, and the enduring impact of incarceration on earnings persists over the long term [18, 19]. Financial instability combined with housing discrimination related to criminal legal history further limits access to stable and safe housing [20, 21]. Strained family relationships further complicate the re‐entry process [22, 23]. Re‐entry difficulty encompasses more than just legal and social risk factors; studies have shown that poor health further complicates the reentry path for those with a history of legal involvement [24–27].

Given the disproportionate burden of disease and criminal legal involvement experienced by African Americans, there is a dearth of research on the independent relationship between postrelease social factors and health outcomes. This study is aimed at examining the relationship between postrelease criminal justice factors (length of incarceration, count of incarceration, and current correctional supervision status) and social risk factors (neighborhood safety, healthcare access, food insecurity, housing insecurity, and social cohesion) and glycemic control in this population. We hypothesized that both postrelease factors (including length and frequency of incarceration and current correctional supervision) and social/environmental risk factors would be associated with inadequate glycemic control in African Americans with Type 2 diabetes and a history of criminal legal involvement.

## 2. Methods

### 2.1. Study Design, Sample, and Setting

This cross‐sectional analysis included 171 African American adults with Type 2 diabetes mellitus and prior exposure to the criminal legal system. Participants were recruited using a multisource strategy that included clinical record screening, peer referral sampling, and targeted outreach in community‐based and re‐entry support settings such as clinics, shelters, faith‐based organizations, and food assistance programs in the greater Milwaukee area within the Midwest region of the United States. Eligibility criteria required participants to be adults aged 18 years or older who self‐identified as Black or African American, had a clinical diagnosis of T2DM, and reported prior criminal legal system involvement and were able to communicate in English. Exclusion criteria included signs of mental confusion—observable cognitive impairment or disorientation that would interfere with the participant′s ability to understand study procedures, provide informed consent, or reliably complete the interview questionnaire at screening—or active psychosis or acute mental disorder during the screening assessment or being under house arrest or continuous electronic monitoring. The data for this study were collected between September 2020 and October 2023. We obtained ethical approval from the institutional review board at the Medical College of Wisconsin (Approval Number PRO34908) before commencing our research, and participants provided informed consent prior to having any data collected about them.

### 2.2. Outcome Measures

Hemoglobin A1c (HbA1c) was the primary outcome variable and was treated as a continuous variable in the analysis. Serum samples were collected and sent to the laboratory for testing, where HbA1c values were obtained.

### 2.3. Primary Independent Variables

#### 2.3.1. Criminal Justice System Factors

These factors include the correctional supervision status and the count of previous incarcerations. These factors were identified from the literature [28]. The current correctional supervision status, reported by individuals, had response options of “yes,” “no,” or “I do not know.” The “yes” response was coded as “yes,” and the “no” and “I do not know” responses were coded as “no.” Similarly, the count of prior incarcerations was self‐reported. The count of prior incarcerations was treated as a continuous variable.

#### 2.3.2. Social Risk/Environmental Factors

These factors include health care access, food security, housing security, neighborhood safety, and social cohesion.

##### 2.3.2.1. Health Care Access

Healthcare access was assessed using a validated BRFSS‐derived insurance coverage indicator capturing self‐reported access to any form of public or private health insurance. For this study we used the question “Do you have any kind of healthcare coverage, including health insurance, prepaid plans such as HMOs, or government plans such as Medicare or Indian Health Service?” to capture health care access. Individuals who indicated yes were coded as having healthcare access.

##### 2.3.2.2. Food Insecurity

Food insecurity was measured using the validated 6‐item USDA Household Food Security Module, which captures severity of resource‐limited food access over the past 12 months [29, 30]. Respondents were asked a series of questions regarding their experiences over the past 12 months, including whether they had enough money to purchase food, if they had to reduce meals or consume less due to financial constraints, if they could afford balanced meals, and if they refrained from eating more despite feeling hungry due to financial limitations [29, 30]. The score on this scale increases with the frequency of these experiences, with higher scores indicating greater levels of food insecurity. For this analysis, the score was dichotomized; a score of 0 or 1 was considered *food secure*, and a score of 2 through 6 considered *food insecure*.

##### 2.3.2.3. Housing Insecurity

Housing insecurity was assessed using items from the BRFSS Social Context Module and defined based on reported financial strain related to rent, mortgage, or utility payments and/or inability to meet housing‐related expenses. We determined the presence of housing insecurity based on these questions, “How often in the past 12 months would you say that you were worried or stressed about having enough money to pay your rent/mortgage?” Response options were “rarely,” “sometimes,” “usually,” or “always” were categorized as “yes” and “never” categorized as “no.” “During the last 12 months, was there a time when you were not able to pay your mortgage, rent or utility bills?” with response type “yes” or “no.” If respondents answered “yes” to any of the four questions, individuals were considered to have housing insecurity.

##### 2.3.2.4. Neighborhood Safety

Neighborhood safety and social cohesion were evaluated using validated multi‐item scales assessing perceived safety, trust, and neighborhood connectedness, with higher scores indicating more adverse perceptions or lower cohesion. “I feel safe walking in my neighborhood during the evening,” “My neighborhood is safe from crime,” and “Violence is a problem in my neighborhood” [31]. The response categories range from 1 (*strongly agree*) to 5 (*strongly disagree*). The score was summed up and treated as a continuous variable.

##### 2.3.2.5. Social Cohesion

Social cohesion was measured using the 5‐item Sampson scale, which include “This is a close‐knit or unified neighborhood,” “People around here are willing to help their neighborhoods,” “People in this neighborhood generally do not get along with each other,” “People in this neighborhood can be trusted,” and “People in this neighborhood do not share the same values.” Responses on each scale vary from 1 (*strongly agree*) to 5 (*strongly disagree*). Sampson scale [32] evaluates participants′ capacity to trust and connect with individuals in their local community. Social cohesion was used as a continuous variable.

### 2.4. Demographic/Comorbidities Measures

Demographic characteristics of the respondents included age, sex, years of education, income, employment status, marital status, BMI, duration of diabetes, comorbidity, and type of medication. Age was treated as a continuous variable. Sex was categorized as male or female. Education was defined by the years of education beyond kindergarten respondents completed and was examined as a continuous variable. Annual income was categorized into less than $25,000 and $25,000 or more. Employment status was dichotomized as either not employed or employed. Marital status was categorized as never married or married/separated/divorced/widowed. BMI was computed by dividing the weight of study participants in kilograms by the square of their height in meters and was examined as a continuous variable. The duration of diabetes was computed by subtracting the age of diabetes diagnosis from the current age of the participant and treated as a continuous variable. Medical comorbidities included hypertension, hyperlipidemia, myocardial infarction, angina, stroke, asthma, cancer, COPD, connective tissue disease, depression, kidney disease, end‐stage renal disease, and leg amputation. These medical comorbidities were self‐reported with response options, “yes,” “no,” or “I do not know.” Each comorbidity was dichotomized, where “yes” responses were coded as “yes,” and responses of “no” or “I do not know” were coded as “no.” The summation of the “yes” responses was used to compute the comorbidity count, and it was analyzed as a continuous variable. The type of diabetes medication was categorized as oral/pill, insulin injectable medication/shot, or a combination of these.

### 2.5. Statistical Analyses

After confirming the normal distribution of variables, we conducted three sets of analyses to explore the relationship between postrelease factors and glycemic control. First, we determined the means and percentages for sociodemographic variables, independent and dependent variables. Second, we examined the independent relationship of (1) criminal justice factors, (2) social risk factors, and (3) demographic/clinical factors with glycemic control using three linear regression models. For the criminal justice factor model, the length of incarceration, count of prior incarcerations, and current supervision status were added as primary independent variables with glycemic control as the outcome. For the social risk factor model, access to healthcare, food insecurity, housing insecurity, social cohesion, and neighborhood safety were added as primary independent variables with glycemic control as the outcome. For the demographic/clinical factor model, age, sex, education, income, employment status, marital status, insurance type, comorbidity, BMI, medication, and duration of diabetes were added as primary independent variables with glycemic control as the outcome. Third, we employed stepwise linear regression with forward selection to examine the association between all variables tested in the initial models and glycemic control (HbA1c). Demographic/clinical variables (age, sex, education, income, employment status, marital status, insurance type, comorbidity, BMI, medication, and duration of diabetes), criminal justice variables (current criminal supervision status, length of incarceration, and count of prior incarcerations), and social risk variables (healthcare access, food insecurity, housing insecurity, social cohesion, and neighborhood safety) were added into the model with glycemic control as the outcome. These variables were incorporated into the final model based on a retention criterion of *p* value of ≤ 0.20. All analyses were performed using STATA Version 17 (Stata Corp LP, College Station, Texas). Statistical significance was set at a two‐sided *p* value of less than 0.05.

## 3. Results

Table 1 shows demographic characteristics for our sample. The sample population had an average age of 57, and approximately 61% were male. A significant portion of the participants were unemployed (81.4%). About 55% of the sample population were unmarried, 87.6% had an annual income of less than $25,000, and 44.3% used oral hypoglycemic agents. The average educational attainment for this group was 12 years, with a mean diabetes duration of 17 years. The average number of comorbidities was 4, and the average BMI was 35 kg/m^2^.

**Table 1:** Sample characteristics for the study sample of African American adults with criminal legal involvement and Type 2 diabetes mellitus (*n* = 171).

	% or *m* *e* *a* *n* ± *s* *t* *a* *n* *d* *a* *r* *d* *d* *e* *v* *i* *a* *t* *i* *o* *n*
**Age**	57.2 ± 8.3
**Sex**	
Male	60.6%
Female	39.4%
**Years of education**	12.5 ± 2.6
**Income**	
< $25,000	87.6%
≥ $25,000	12.4%
**Employment status**	
Not employed	81.4%
Employed	18.6%
**Marital status**	
Never married	55.3%
Married/separated/divorced/widowed	44.7%
**Body mass index (BMI)**	35.5 ± 8.6
**Duration of diabetes mellitus**	17.8 ± 13.6
**Comorbidity**	4.2 ± 2.2
**Antidiabetes medication**	
Pill or oral	44.3%
Insulin, shot or injectable	22.2%
Both	33.5%

Table 2 shows an overview of the average values and frequencies for the primary independent and dependent variables. Within our sample population, the average HbA1c was 8.0%. About 5.8% of respondents reported being currently under correctional supervision. The average number of incarcerations since the age of 18 was 6.4, with an average incarceration length of 29.8 months. About 95% of the study population reported they had access to healthcare. In addition, 48% reported food insecurity, and 46.2% reported housing insecurity.

**Table 2:** Summary of postrelease variables and glycemic control (*n* = 171).

Variables	% or mean ± S.D (*n* = 171)
**Criminal justice system factors**	
Probation status	5.9%
Number of incarcerations	6.4 ± 10.3
Length of incarceration (in months)	29.8 ± 62.5
**Social risk factors**	
Healthcare access	94.7%
Food insecurity	47.9%
Housing insecurity	47.9%
Neighborhood safety	8.9 ± 2.2
Social cohesion	15.0 ± 2.9

*Note:* Range of scores for social cohesion: 5.0–25.0; neighborhood safety: 3.0–15.0.

Table 3 shows partially adjusted linear regression and stepwise regression models. In the partially adjusted models, there was no significant relationship between the criminal justice factors and glycemic control. In the relationship between social risk/environmental factors and glycemic control, respondents who experienced housing insecurity (*β*‐coefficient: 0.93; 95% CI: 0.26, 1.60) had higher HbA1c compared with those with no housing insecurity. In the relationship between demographic/clinical factors and glycemic control, age (*β*‐coefficient: −0.05; 95% CI: −0.09, −0.003) and comorbidity (*β*‐coefficient: −0.25; 95% CI: −0.40, −0.10) were associated with lower HbA1c, which implies older participants and those with more comorbidities tended to have better glycemic control. Compared with respondents taking oral hypoglycemic agents only, those using insulin (*β*‐coefficient: 0.93; 95% CI: 0.09, 1.77) and those using both insulin and oral hypoglycemic agents (*β*‐coefficient: 1.36; 95% CI: 0.65, 2.07) were associated with higher HbA1c. In the fully adjusted stepwise regression model, age, housing insecurity, and length of incarceration though retained in the model, were not significantly associated with HbA1c. However, use of insulin alone (*β*‐coefficient: 0.95; 95% CI: 0.16, 1.73) and a combination of insulin and oral hypoglycemic agents (*β*‐coefficient: 1.44; 95% CI: 0.77, 2.12) were significantly associated with higher HbA1c. Additionally, higher comorbidity count (*β*‐coefficient: −0.25; 95% CI: −0.40, −0.11) was significantly associated with lower HbA1c. Finally, compared with individuals with private/military/other insurance, those with Medicare (*β*‐coefficient: 1.40; 95% CI: 0.43, 2.37) and Medicaid (*β*‐coefficient: 1.11; 95% CI: 0.12, 2.10) had higher HbA1c.

**Table 3:** Adjusted relationship between postrelease factors and glycemic control.

	Partially adjusted *β* (95% CI)	Stepwise regression *β* (95% CI)
**Criminal justice factors**		
Probation status		
No	Reference	
Yes	−0.08 (−1.57, 1.40)	
Number of incarcerations	−0.002 (−0.03, 0.03)	
Length of recent incarcerations	−0.01 (−0.01, 0.001)	−0.01 (−0.01; −0.001)
**Social risk factors**		
Health care access		
No	Reference	
Yes	−0.003 (−1.44, 1.43)	
Food insecurity		
No	Reference	
Yes	0.23 (−0.45, 0.92)	
Housing insecurity		
No	Reference	Reference
Yes	**0.93 (0.26, 1.60)** ^∗∗^	0.46 (−0.16, 1.08)
Neighborhood safety	0.09 (−0.05, 0.23)	
Social cohesion	−0.04 (−0.15, 0.08)	
**Demographics/clinical factors**		
Age	−**0.05 (**−**0.09,** −**0.003)** ^∗^	−0.03 (−0.07, 0.01)
Sex		
Female	Reference	
Male	0.07 (−0.61, 0.74)	
Education	0.01 (−0.12, 0.13)	
Income		
< $25,000	Reference	
> $25,000	−0.11 (−1.15, 0.92)	
Insurance type		
Private/military/other	Reference	
Medicare	1.09 (−0.05, 2.23)	**1.40 (0.43, 2.37)** ^∗∗^
Medicaid	0.78 (−0.40, 1.96)	**1.11 (0.12, 2.10)** ^∗^
Employment status	−0.30 (−1.28, 0.68)	
Marital status		
Never married	Reference	
Married/separated/divorced/widowed	0.07 (−0.56, 0.71)	
Body mass index (BMI)	0.003 (−0.03, 0.04)	
Duration of diabetes	0.01 (−0.02, 0.03)	
Comorbidity	−**0.25 (**−**0.40,** −**0.10)** ^∗∗∗^	−**0.25 (**−**0.39,** −**0.11)** ^∗∗∗^
Antidiabetes medication		
Pill or oral	Reference	
Insulin, shot or injectable	**0.93 (0.09, 1.77)** ^∗^	**0.94 (0.16, 1.72)** ^∗^
Both	**1.36 (0.65, 2.07)** ^∗∗∗^	**1.44 (0.77, 2.12)** ^∗∗∗^

*Note:* Bold indicates statistical significance. Empty boxes indicate variables dropped from the model based on stepwise regression.

Abbreviation: CI, confidence interval.

∗∗∗*p* < 0.001, ∗∗*p* < 0.01, ∗*p* < 0.05.

## 4. Discussion

In our sample population of African American adults with a history of criminal legal involvement and T2DM, postrelease criminal justice factors and social risk were not independently associated with glycemic control. However, in the stepwise regression, some demographic/clinical factors were significantly associated with glycemic control. Compared with the use of oral hypoglycemic agents alone, the use of insulin alone and a combination of insulin and oral hypoglycemic agents were associated with a 0.9% and 1.4% increase in HbA1c level, respectively. Every unit increase in comorbidity count was associated with a 0.25% decrease in HbA1c level. Compared with those with private/military/other insurance, individuals with Medicare and Medicaid were associated with a 1.4% and 1.1% increase in HbA1c level. Notably, the coexistence of high BMI and food insecurity within our study population may indicate limited access to affordable, nutritious foods and a reliance on calorie‐dense, nutrient‐poor options, along with other structural factors that could adversely affect glycemic control.

To the best of our knowledge, this is one of the first studies that examines the relationship between postrelease criminal justice factors or social risk/environmental factors and glycemic control among African Americans with T2DM and history of criminal legal involvement. Interestingly, in stepwise regression models neither criminal justice factors nor social risk factors were significantly associated with glycemic control. Instead, demographic and clinical factors were significantly associated, suggesting that factors such as insurance type, comorbidity, and medication may play a more significant role in influencing glycemic control in this population. These findings underscore the complex interplay of demographic and clinical factors in health outcomes and highlight the need for further research to better understand and address health outcomes within this population. Future research should expand the population of postrelease individuals to allow investigation into whether factors associated with poor postrelease diabetes outcomes vary by race/ethnicity.

In our study, African Americans with history of criminal legal involvement and T2DM who reported being on Medicare and Medicaid had 1.40% and 1.11% increase in HbA1c compared with those who had private/military/other insurance. Our result is consistent with prior research findings [32, 33]. However, our results are in contrast with a study that used the 2020 Behavioral Risk Factor Surveillance System data [34], which found minimal variations in HbA1c across Medicare, Medicaid, Department of Veterans Affairs, and private insurance [34]. As in our study, the majority of those that had a history of criminal legal involvement suffered from collateral consequences of incarceration such as unemployment and low income [35, 36], and therefore upon release from jail or prison, majority were insured under Medicaid [37]. Another study conducted in 2009 in US community health centers found that patients with varying types of insurance may experience different levels of care quality, leading to distinct health outcomes [33]. The lack of continuity of care among Medicaid patients may also be a significant contributor to poor glycemic control in this population group [38].

Furthermore, our study revealed a significant relationship between clinical factors (comorbidity count and medication type) and HbA1c. Each additional comorbidity was associated with a 0.25% decrease in HbA1c, which is consistent with prior literature [39, 40]. A possible explanation is that individuals with multiple comorbidities may have more frequent contact with healthcare systems, resulting in closer monitoring, greater medication adjustment, and improved diabetes management. Additionally, patients with more chronic illnesses may receive more multidisciplinary care and be more engaged in ongoing treatment. Our study also showed that respondents on insulin or injectables had worse glycemic control compared with those on oral agents, which is again consistent with prior findings [41–44]. A possible explanation for these findings is that most individuals with poor glycemic control or with multiple comorbidities are usually prescribed insulin and so insulin use might be a surrogate for the poor glycemic control [45]. Similarly, the relationship between insulin use and poor glycemic control could be attributed to poor self‐efficacy and lack of knowledge about diabetes, insulin dosage, and glycemic levels [46].

Although some states in the United States have implemented Medicaid expansion to ensure the provision of health insurance for everyone, including those with a history of criminal legal involvement [47], our findings indicate the need for increasing research and policy considerations regarding the potential impact of a patient’s insurance type on the quality of healthcare received. Our study specifically focused on African Americans, and it would be pertinent to explore the potential interaction between race and insurance type. Consequently, future research and policies should scrutinize racial disparities in the correlation between insurance type and glycemic control or health outcomes among individuals with a history of criminal legal involvement and T2DM. Although the association between comorbidity count and HbA1c was statistically significant, the magnitude of change per additional comorbidity may be modest from a clinical standpoint. Future interventions should target individuals with fewer comorbidities who have a history of criminal legal involvement and T2DM, as they are less likely to utilize healthcare resources for optimizing their blood glucose levels [48, 49].

Our study acknowledges some limitations. First, it is important to note that respondents self‐reported their experiences postrelease from incarceration. This has the potential of introducing social desirability bias with the possibility of underreporting; however, validated questions were used to increase the reliability of results. Future studies that collect more detailed incarceration history should use a variety of ways to measure incarceration to understand potential influence on outcomes. Second, given the cross‐sectional design, temporal and causal inferences cannot be established from these associations. Third, the modest sample size may have reduced statistical power to detect smaller effect sizes and interaction effects. Stepwise regression was used to minimize the impact of a small sample size; however, as more studies are conducted collection of additional variables, such as measurement of complications of diabetes or investigation into individual familial structure or detailed neighborhood factors can be investigated with larger samples. Fourth, the limited geographic location of respondents and exclusion of mental illness limit generalizability to all postrelease individuals. Larger studies that cover multiple geographic regions and follow individuals over time will be important to further inform research in this field. Despite these limitations, it is worth noting that our study holds strengths, particularly in the use of a comprehensive set of validated survey measures to collect information on SDOH.

## 5. Conclusion

In our study, postrelease criminal justice factors and social risk factors were not associated with glycemic control in African Americans with T2DM and a history of criminal legal involvement. However, demographic/clinical factors including comorbidity, type of medication, and insurance type were associated with glycemic control in our sample population. Future research should further evaluate the relationship between insurance type and glycemic control in individuals with a history of criminal legal involvement and T2DM. Additionally, future interventions should target adults with fewer comorbidities who are less likely to utilize healthcare resources for their glycemic control.

## Author Contributions

O.A.: data curation, investigation, methodology, project administration, writing—original draft, and review and editing. R.J.W.: methodology, supervision, and writing—review and editing. J.A.C.: methodology and writing—review and editing. L.H.: methodology and writing—review and editing. J.S.W.: methodology and writing—review and editing. L.E.E.: conceptualization, formal analysis, methodology, supervision, validation, and writing—review and editing.

## Funding

This study was supported by the National Institute of Diabetes and Digestive Kidney Disease (R01DK118038, R01DK120861, R21DK123720, K01DK131319, K23DK132505) and National Institute for Minority Health and Health Disparities (R01MD013826, R01MD018012, R01MD017574).

## Disclosure

All authors read and approved the final version of the manuscript. L.E.E. served as the manuscript guarantor and had full access to all study data and takes responsibility for the integrity of the data and the accuracy of the data analysis.

## Conflicts of Interest

The authors declare no conflicts of interest.

## Data Availability

The data that support the findings of this study are available from the corresponding author, L.E.E., upon reasonable request.

## References

[1] American Diabetes Association , Statistics About Diabetes, 2023, American Diabetes Association, Retrieved from: https://diabetes.org/about-diabetes/statistics/about-diabetes.

[2] Centers for Disease Control and Prevention , National Diabetes Statistics Report, 2023. Atlanta, GA: Centers for Disease Control and Prevention, U.S. Dept of Health and Human Services, 2023, Centers for Disease Control and Prevention, Retrieved from: https://www.cdc.gov/diabetes/health-equity/diabetes-by-the-numbers.html.

[3] Centers for Disease Control and Prevention , National Diabetes Statistics Report, 2020. Atlanta, GA: Centers for Disease Control and Prevention, U.S. Dept of Health and Human Services, 2020, Centers for Disease Control and Prevention.

[4] Walker R. J. , Smalls B. L. , Campbell J. A. , Strom Williams J. L. , and Egede L. E. , Impact of Social Determinants of Health on Outcomes for Type 2 Diabetes: A Systematic Review, Endocrine, 2014, 47, no. 1, 29–48, 10.1007/s12020-014-0195-0, 24532079. PMC7029167 24532079

[5] Hill-Briggs F. , Adler N. E. , Berkowitz S. A. , Chin M. H. , Gary-Webb T. L. , Navas-Acien A. , Thornton P. L. , and Haire-Joshu D. , Social Determinants of Health and Diabetes: A Scientific Review, Diabetes Care. (2020) 44, no. 1, 258–279, 10.2337/dci20-0053, 33139407. 33139407 PMC7783927

[6] World Health Organization , Commission on Social Determinants of Health, Closing the Gap in a Generation: Health Equity Through Action on the Social Determinants of Health. CSDH Final Report. (2008) WHO.

[7] Solar O. and Irwin A. , A Conceptual Framework for Action on the Social Determinants of Health, Social Determinants of Health Discussion Paper 2 (Policy and Practice) World Health Organization, 2010, WHO Document Production Services.

[8] Walker R. J. , Garacci E. , Palatnik A. , Ozieh M. N. , and Egede L. E. , The Longitudinal Influence of Social Determinants of Health on Glycemic Control in Elderly Adults With Diabetes, Diabetes Care. (2020) 43, no. 4, 759–766, 10.2337/dc19-1586, 32029639. 32029639 PMC7085811

[9] Hill-Briggs F. and Fitzpatrick S. L. , Overview of Social Determinants of Health in the Development of Diabetes, Diabetes Care. (2023) 46, no. 9, 1590–1598, 10.2337/dci23-0001, 37354331. 37354331

[10] Alderwick H. and Gottlieb L. M. , Meanings and Misunderstandings: A Social Determinants of Health Lexicon for Health Care Systems, Milbank Quarterly. (2019) 97, no. 2, 407–419, 10.1111/1468-0009.12390, 31069864. 31069864 PMC6554506

[11] Becker S. and Alexander L. , Understanding the Impacts of Incarceration on Health, ReThink Health. (2016) 11, Available at: https://rethinkhealth.org/wp-content/uploads/2016/04/ReThink-Health-March-17-Report-1.pdf.

[12] Nowotny K. M. and Kuptsevych-Timmer A. , Health and Justice: Framing Incarceration as a Social Determinant of Health for Black Men in the United States, Sociol Compass. (2018) 12, no. 3, e12566, 10.1111/soc4.12566.

[13] Massoglia M. and Remster B. , Linkages Between Incarceration and Health, Public Health Reports. (2019) 134, no. 1_Supplement, 8S–14S, 10.1177/0033354919826563, 31059410. 31059410 PMC6505320

[14] The Sentencing Project , The Color of Justice: The Racial and Ethnic Disparity in State Prisons, 2021, The Sentencing Project, Assessed at: https://s3.documentcloud.org/documents/21084578/the-color-of-justice-racial-and-ethnic-disparity-in-state-prisons. pdf.

[15] Travis J. , But They All Come Back: Facing the Challenges of Prisoner Reentry, 2005, Urban Institute Press.

[16] Petersilia J. , When Prisoners Come Home: Parole and Prisoner Reentry, 2003, 67, no. 3, Oxford University Press, Reviewed in: Fed Probation 10.1093/acprof:oso/9780195160864.001.0001.

[17] Liem M. and Weggemans D. , Reintegration Among High-Profile Ex-Offenders, Journal of Developmental and Life-Course Criminology. (2018) 4, no. 4, 473–490, 10.1007/s40865-018-0093-x, 30873339. 30873339 PMC6390714

[18] Western B. , The Impact of Incarceration on Wage Mobility and Inequality, American Sociological Review. (2002) 67, no. 4, 526–546, 10.2307/3088944.

[19] Western B. , Kling J. R. , and Weiman D. F. , The Labor Market Consequences of Incarceration, Crime & Delinquency. (2001) 47, no. 3, 410–427, 10.1177/0011128701047003007.

[20] Geller A. and Curtis M. A. , A Sort of Homecoming: Incarceration and the Housing Security of Urban Men, Social Science Research. (2011) 40, no. 4, 1196–1213, 10.1016/j.ssresearch.2011.03.008, 21927519. 21927519 PMC3173782

[21] Metraux S. , Roman C. G. , and Cho R. S. , Incarceration and Homelessness, Toward Understanding Homelessness, The 2007 National Symposium on Homelessness Research.

[22] Braman D. , Doing Time on the Outside: Incarceration and Family Life in Urban America, 2004, University of Michigan Press, 10.3998/mpub.17629.

[23] Massoglia M. , Remster B. , and King R. D. , Stigma or Separation? Understanding the Incarceration-Divorce Relationship, Social Forces. (2011) 90, no. 1, 133–155, 10.1093/sf/90.1.133.

[24] Binswanger I. A. , Stern M. F. , Deyo R. A. , Heagerty P. J. , Cheadle A. , Elmore J. G. , and Koepsell T. D. , Release From Prison--a High Risk of Death for Former Inmates, New England Journal of Medicine. (2007) 356, no. 2, 157–165, 10.1056/NEJMsa064115. 17215533 PMC2836121

[25] Fazel S. and Baillargeon J. , The Health of Prisoners, Lancet. (2011) 377, no. 9769, 956–965, 10.1016/S0140-6736(10)61053-7. 21093904

[26] Schnittker J. and John A. , Enduring Stigma: The Long-Term Effects of Incarceration on Health, Journal of Health and Social Behavior. (2007) 48, no. 2, 115–130, 10.1177/002214650704800202, 17583269. 17583269

[27] Massoglia M. and Pridemore W. A. , Incarceration and Health, Annual Review of Sociology. (2015) 41, 291–310, 10.1146/annurev-soc-073014-112326, 30197467. PMC6124689 30197467

[28] Binswanger I. A. , Krueger P. M. , and Steiner J. F. , Prevalence of Chronic Medical Conditions Among Jail and Prison Inmates in the USA Compared With the General Population, Journal of Epidemiology & Community Health. (2009) 63, no. 11, 912–919, 10.1136/jech.2009.090662, 19648129. 19648129

[29] Bickel G. , Nord M. , Price C. , Hamilton W. , and Cook J. , Measuring Food Security in the United States: Guide to Measuring Household Food Security, 2000, Research in Agricultural and Applied Economics.

[30] Blumberg S. J. , Bialostosky K. , Hamilton W. L. , and Briefel R. R. , The Effectiveness of a Short Form of the Household Food Security Scale, American Journal of Public Health. (1999) 89, no. 8, 1231–1234, 10.2105/ajph.89.8.1231, 10432912. 10432912 PMC1508674

[31] Echeverria S. E. , Diez-Roux A. V. , and Link B. G. , Reliability of Self-Reported Neighborhood Characteristics, Journal of Urban Health. (2004) 81, no. 4, 682–701, 10.1093/jurban/jth151, 15466849. 15466849 PMC3455923

[32] Gold R. S. , Unkart J. T. , McClelland R. L. , Bertoni A. G. , and Allison M. A. , Health Insurance Status and Type Associated With Varying Levels of Glycemic Control in the US: The Multi-Ethnic Study of Atherosclerosis (MESA), Primary Care Diabetes. (2021) 15, no. 2, 378–384, 10.1016/j.pcd.2020.11.011, 33309035. 33309035 PMC7936947

[33] Zhang X. , Bullard K. M. , Gregg E. W. , Beckles G. L. , Williams D. E. , Barker L. E. , Albright A. L. , and Imperatore G. , Access to Health Care and Control of ABCs of Diabetes, Diabetes Care. (2012) 35, no. 7, 1566–1571, 10.2337/dc12-0081, 22522664. 22522664 PMC3379598

[34] Nelson K. M. , Chapko M. K. , Reiber G. , and Boyko E. J. , The Association Between Health Insurance Coverage and Diabetes Care; Data From the 2000 Behavioral Risk Factor Surveillance System, Health Services Research. (2005) 40, no. 2, 361–372, 10.1111/j.1475-6773.2005.00361.x, 15762896. 15762896 PMC1361145

[35] Khan M. R. , Behrend L. , Adimora A. A. , Weir S. S. , White B. L. , and Wohl D. A. , Dissolution of Primary Intimate Relationships During Incarceration and Implications for Post-Release HIV Transmission, Journal of Urban Health. (2011) 88, no. 2, 365–375, 10.1007/s11524-010-9538-1, 21286825. 21286825 PMC3079034

[36] Binswanger I. A. , Nowels C. , Corsi K. F. , Glanz J. , Long J. , Booth R. E. , and Steiner J. F. , Return to Drug Use and Overdose After Release From Prison: A Qualitative Study of Risk and Protective Factors, Addiction Science & Clinical Practice. (2012) 7, no. 1, 10.1186/1940-0640-7-3, 22966409. PMC3414824 22966409

[37] National Conference of State Legislatures , Connecting Recently Released Prisoners to Health Care: How to Leverage Medicaid, 2016, National Conference of State Legislatures, Accessed May 21, 2026. Available from: National Conference of State Legislatures.

[38] Bindman A. B. , Chattopadhyay A. , and Auerback G. M. , Interruptions in Medicaid Coverage and Risk for Hospitalization for Ambulatory Care–Sensitive Conditions, Annals of Internal Medicine. (2008) 149, no. 12, 854–860, 10.7326/0003-4819-149-12-200812160-00005, 19075205. 19075204

[39] Bralić Lang V. and Bergman Marković B. , Prevalence of Comorbidity in Primary Care Patients With Type 2 Diabetes and Its Association With Elevated HbA1c: A Cross-Sectional Study in Croatia, Scandinavian Journal of Primary Health Care. (2016) 34, no. 1, 66–72, 10.3109/02813432.2015.1132886, 26853192. 26853192 PMC4911025

[40] Vitry A. I. , Roughead E. E. , Preiss A. K. , Ryan P. , Ramsay E. N. , Gilbert A. L. , Caughey G. E. , Shakib S. , Esterman A. , Zhang Y. , and McDermott R. A. , Influence of Comorbidities on Therapeutic Progression of Diabetes Treatment in Australian Veterans: A Cohort Study, PLoS One. (2010) 5, no. 11, e14024, 10.1371/journal.pone.0014024, 21103337. 21103337 PMC2984440

[41] Mamo Y. , Bekele F. , Nigussie T. , and Zewudie A. , Determinants of Poor Glycemic Control Among Adult Patients With Type 2 Diabetes Mellitus in Jimma University Medical Center, Jimma Zone, Southwest Ethiopia: A Case Control Study, BMC Endocrine Disorders. (2019) 19, no. 1, 10.1186/s12902-019-0421-0, 31464602. PMC6716911 31464602

[42] Espinosa M. M. , Almeida V. R. , and Nascimento V. F. , Poor Glycemic Control and Associated Factors in Diabetic People Attending a Reference Outpatient Clinic in Mato Grosso, Brazil, Investigación y Educación en Enfermería. (2021) 39, no. 3, 10.17533/udea.iee.v39n3e10, 34822237. PMC8912167 34822237

[43] Venkatraman S. , Echouffo-Tcheugui J. B. , Selvin E. , and Fang M. , Trends and Disparities in Glycemic Control and Severe Hyperglycemia Among US Adults With Diabetes Using Insulin, 1988-2020, JAMA Network Open. (2022) 5, no. 12, e2247656, 10.1001/jamanetworkopen.2022.47656, 36538330. 36538330 PMC9856837

[44] Suprapti B. , Izzah Z. , Anjani A. G. , Andarsari M. R. , Nilamsari W. P. , and Nugroho C. W. , Prevalence of Medication Adherence and Glycemic Control Among Patients With Type 2 Diabetes and Influencing Factors: A Cross-Sectional Study, Global Epidemiology. (2023) 5, 100113, 10.1016/j.gloepi.2023.100113, 37638377. 37638377 PMC10446000

[45] Dinavari M. F. , Sanaie S. , Rasouli K. , Faramarzi E. , and Molani-Gol R. , Glycemic Control and Associated Factors Among Type 2 Diabetes Mellitus Patients: A Cross-Sectional Study of Azar Cohort Population, BMC Endocrine Disorders. (2023) 23, no. 1, 10.1186/s12902-023-01515-y, 38087260. PMC10714613 38087260

[46] Tong W. T. , Vethakkan S. R. , and Ng C. J. , Why Do Some People With Type 2 Diabetes Who Are Using Insulin Have Poor Glycaemic Control? A Qualitative Study, BMJ Open. (2015) 5, no. 1, e006407, 10.1136/bmjopen-2014-006407, 25633285. PMC4316456 25633285

[47] Jannetta J. , Dorn S. , Kurs E. , Reginal T. , Marks J. , Serafi K. , Guyer J. , and Cantrell C. , Strategies for Connecting Justice-Involved Populations to Health Coverage and Care, 2018, Urban Institute, Retrieved from https://policycommons.net/artifacts/631177/strategies-for-connecting-justice-involved-populations-to-health-coverage-and-care/1612556.

[48] Struijs J. N. , Baan C. A. , Schellevis F. G. , Westert G. P. , and van den Bos G. A. , Comorbidity in Patients With Diabetes Mellitus: Impact on Medical Health Care Utilization, BMC Health Services Research. (2006) 6, 10.1186/1472-6963-6-84, 16820048. PMC1534031 16820048

[49] Sampson R. J. , Raudenbush S. W. , and Earls F. , Neighborhoods and Violent Crime: A Multilevel Study of Collective Efficacy, Science. (1997) 277, no. 5328, 918–924, 10.1126/science.277.5328.918. 9252316

